# Diverse patterns of genomic targeting by transcriptional regulators in *Drosophila melanogaster*

**DOI:** 10.1101/gr.168807.113

**Published:** 2014-07

**Authors:** Matthew Slattery, Lijia Ma, Rebecca F. Spokony, Robert K. Arthur, Pouya Kheradpour, Anshul Kundaje, Nicolas Nègre, Alex Crofts, Ryan Ptashkin, Jennifer Zieba, Alexander Ostapenko, Sarah Suchy, Alec Victorsen, Nader Jameel, A. Jason Grundstad, Wenxuan Gao, Jennifer R. Moran, E. Jay Rehm, Robert L. Grossman, Manolis Kellis, Kevin P. White

**Affiliations:** 1 Institute for Genomics & Systems Biology, Department of Human Genetics, The University of Chicago, Chicago, Illinois 60637, USA;; 2 Computer Science and Artificial Intelligence Laboratory (CSAIL), Massachusetts Institute of Technology (MIT), Cambridge, Massachusetts 02139, USA;; 3 Université de Montpellier II and INRA, UMR1333 DGIMI, F-34095 Montpellier, France;; 4 Broad Institute of MIT and Harvard, Cambridge, Massachusetts 02142, USA

## Abstract

Annotation of regulatory elements and identification of the transcription-related factors (TRFs) targeting these elements are key steps in understanding how cells interpret their genetic blueprint and their environment during development, and how that process goes awry in the case of disease. One goal of the modENCODE (model organism ENCyclopedia of DNA Elements) Project is to survey a diverse sampling of TRFs, both DNA-binding and non-DNA-binding factors, to provide a framework for the subsequent study of the mechanisms by which transcriptional regulators target the genome. Here we provide an updated map of the *Drosophila melanogaster* regulatory genome based on the location of 84 TRFs at various stages of development. This regulatory map reveals a variety of genomic targeting patterns, including factors with strong preferences toward proximal promoter binding, factors that target intergenic and intronic DNA, and factors with distinct chromatin state preferences. The data also highlight the stringency of the Polycomb regulatory network, and show association of the Trithorax-like (Trl) protein with hotspots of DNA binding throughout development. Furthermore, the data identify more than 5800 instances in which TRFs target DNA regions with demonstrated enhancer activity. Regions of high TRF co-occupancy are more likely to be associated with open enhancers used across cell types, while lower TRF occupancy regions are associated with complex enhancers that are also regulated at the epigenetic level. Together these data serve as a resource for the research community in the continued effort to dissect transcriptional regulatory mechanisms directing *Drosophila* development.

Whole-genome sequencing has become increasingly straightforward in recent years, though our ability to interpret these genomes is still far from complete. Understanding the regulatory genome—the noncoding portion of the genome dictating where, when, and to what level genes are expressed—remains a significant challenge. Annotation and characterization of regulatory elements is especially important for metazoan organisms, where complex three-dimensional body plans consisting of many cell types are ultimately derived from the genomic blueprint of a single zygote. *Drosophila melanogaster* has been at the forefront of the biology of transcriptional regulation for decades, with polytene chromosome studies providing some of our earliest glimpses into genome-wide gene regulatory and protein–DNA interactions (Ritossa 1964; Ashburner 1967, 1970; Silver and Elgin 1976; Jamrich et al. 1977; Andrew and Scott 1994). *Drosophila* continues and will remain to be a valuable model for developmental gene regulation due to its ease of genetic manipulation and plethora of comparative genomics resources (Slattery et al. 2012).

Several pioneering studies have provided a genome-wide view into aspects of the *Drosophila* regulatory genome; from RNA polymerase II to Polycomb-Response Elements, insulator elements, chromatin states, transcription factors, and even the conformational architecture of nucleus (Nègre et al. 2006, 2010, 2011; Zeitlinger et al. 2007; Kwong et al. 2008; Li et al. 2008; Filion et al. 2010; Kharchenko et al. 2011; Sexton et al. 2012; Slattery et al. 2012; White 2012). Several recent reviews provide an excellent synthesis of many of these studies (Biggin 2011; Delest et al. 2012; Lelli et al. 2012; Ong and Corces 2012; Slattery et al. 2012; Spitz and Furlong 2012; White 2012). Briefly, the picture emerging from these studies is one of a genome organized into distinct chromatin types, roughly separable into ‘active’ and ‘repressive’ states, physically separated from one another within the three-dimensional nucleus; in some cases regulatory functions are located in spatially discrete sites (e.g., transcription factories and Polycomb bodies) (van Steensel 2011; Delest et al. 2012). Within this chromatin environment, binding of transcriptional regulators to DNA is seemingly widespread, at least relative to the expected number of direct regulatory target genes for most TRFs, and often highly overlapping (i.e., highly occupied target [HOT] regions) (Moorman et al. 2006; Li et al. 2008; Nègre et al. 2011). Higher-order chromatin structure and spatially discrete regions of high TRF concentration likely impact TRF-binding patterns genome wide, although the local chromatin landscape is also influential because DNA accessibility is a major determinant of TRF binding (MacArthur et al. 2009; Guertin and Lis 2010; Li et al. 2011b). Though far from complete, annotation of the *Drosophila* regulatory genome has begun thanks to these studies, and focused genetic and genomic approaches are being used to address mechanistic questions stemming from them (Guertin and Lis 2010; Kvon et al. 2012).

The importance of focused studies of gene regulatory networks—studies exploring panels of TRFs working within the same network, or studies exploring the impact of cellular context on TRF–DNA interactions—cannot be overstated. The goal of the modENCODE (model organism ENCyclopedia of DNA Elements) Project, however, is to survey a diverse sampling of transcriptional regulators, both DNA-binding and non-DNA-binding factors, to get a broad view of the patterns by which transcriptional regulators target the genome. Here we provide an updated map of the *Drosophila melanogaster* regulatory genome based on the location of localization of 84 transcriptional regulators at various stages of development.

## Results

We describe in vivo genome-wide binding patterns of 84 *Drosophila* transcriptional regulatory factors (TRFs), using both previously published data sets and new data generated by the modENCODE Project (Li et al. 2008; Zinzen et al. 2009; Nègre et al. 2011; Supplemental Table S1). Overall, 65 DNA-binding proteins and 19 non-DNA-binding proteins (cofactors, chromatin-binding factors) are represented; most factors were tested in one developmental stage or cell line, though multiple factors were tested in multiple contexts (Supplemental Table S1). In total, these data, all of which are available through the modENCODE Data Coordination Center (http://intermine.modencode.org/) or Gene Expression Omnibus (GEO; see Supplemental Table S1 for accession numbers), represent 171 separate genome-wide ChIP experiments performed in duplicate or greater, 413,743 TRF-binding sites, and 50,336 unique TRF-binding regions.

### Genomic features targeted by transcriptional regulators

Gene expression is controlled by regulatory DNA sequences and the TRFs that interact with these sequences. A significant amount of regulatory information is found immediately upstream of transcription start sites in gene promoter regions. However, the complex gene expression patterns of metazoans often require additional, combinatorial input from distal regulatory sequences known as enhancers, or *cis*-regulatory modules (CRMs) (Lelli et al. 2012; Spitz and Furlong 2012). To get an overall view of the genomic features bound by the transcriptional regulators studied, we characterized binding events as falling into one of the following categories: promoter proximal, intergenic, intron, exon, or downstream (Supplemental Fig. S1). The binding of most general regulators (HDACs and chromatin remodelers) is highly biased toward promoter-proximal binding, and the majority of site-specific DNA-binding TRFs also display significant promoter-proximal binding, although there is a substantial subset that prefers distal binding sites. For instance, the developmental regulators Eve, Hth, Pan (also known as dTCF), EcR, and USP (Harding et al. 1986; Yao et al. 1992; Mann et al. 2009; Archbold et al. 2012) all bind intergenic or intronic DNA >50% of the time across multiple developmental stages, suggesting that these factors often act at distal enhancers (Supplemental Fig. S1). Thus, in these cases, global-binding preferences are consistent with TRF molecular function.

### Chromatin types targeted by transcriptional regulators

Chromatin landscape has the potential to significantly influence the binding of transcriptional regulators to DNA, both through local and global influences on DNA accessibility. Two recent genome-wide studies have annotated the *Drosophila* genome based on chromatin state. Despite using different cell types, experimental techniques, and chromatin factors these independent studies generated functionally consistent chromatin state maps (Filion et al. 2010; Roy et al. 2010; Kharchenko et al. 2011); the modENCODE model consists of nine chromatin states, while the Filion et al. (2010) model describes five chromatin states. For simplicity, we focus here on the five-state model, in which chromatin states are assigned colors. YELLOW and RED chromatin are the two ‘active’ states, with the former generally associated with ubiquitous genes and the latter with patterned genes. BLACK, GREEN, and BLUE represent the three ‘inactive’ states; BLACK regions are relatively gene-poor, GREEN is associated with heterochromatin, and BLUE is associated with Pc-mediated silencing. Importantly, although the five-state model is based on data from Kc167 cells, our developmental timecourse of chromatin modifications reveals that many features captured in this cell line are consistent throughout development; for example, BLUE chromatin is always marked with repressive histone modifications and YELLOW and RED chromatin is always associated with active histone modifications (Filion et al. 2010; Nègre et al. 2011; Supplemental Fig. S2). The consistency of these global trends indicates that the five-state model is relevant to many developmental stages. We sought to explore the relationship between these chromatin states and transcriptional regulator binding patterns (Filion et al. 2010). Thus, for each TRF, we looked at the fraction of binding events that fall into each of the five chromatin types.

**Figure 1.**
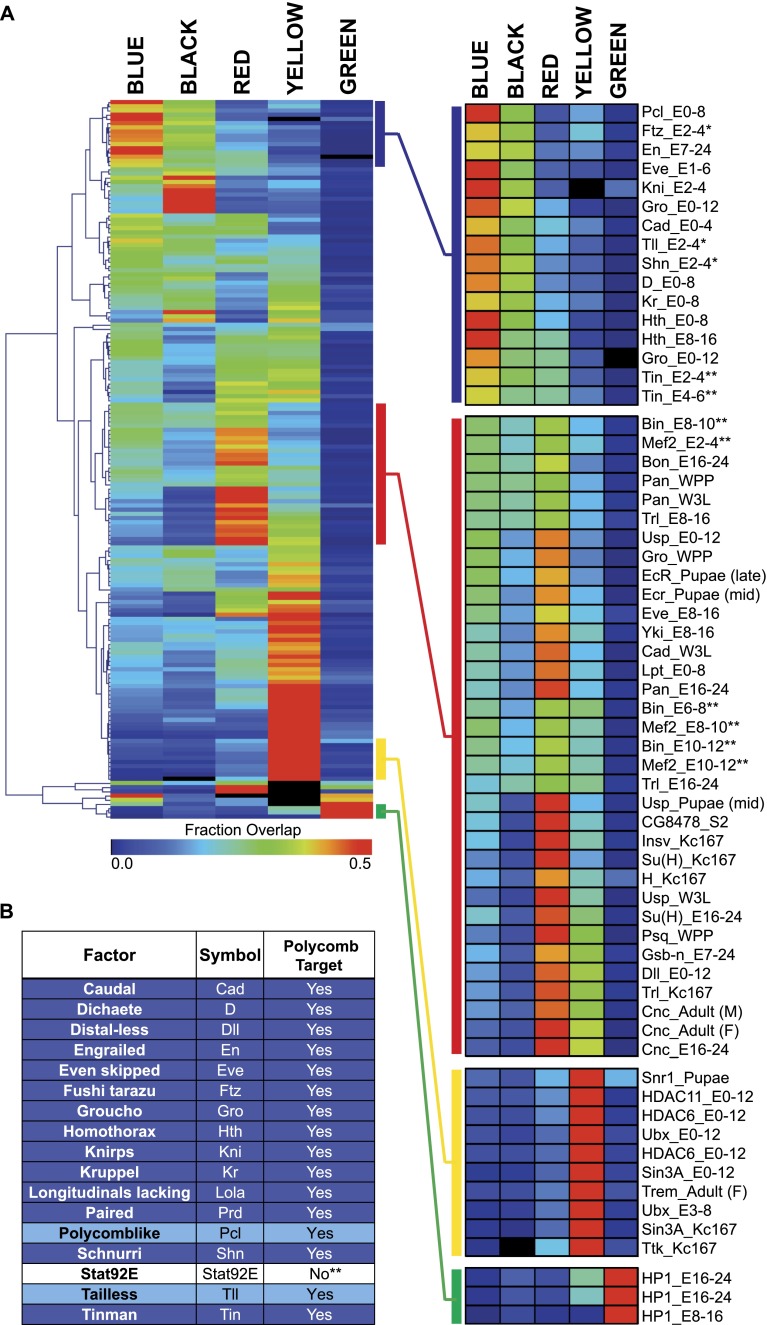
Transcriptional regulator overlap with chromatin states. (*A*) Heatmap representing the fraction of TRF binding overlapping chromatin states as defined by Filion et al. (2010). (*B*) TRFs with >35% binding in BLUE/Pc associated chromatin. TRFs shaded dark blue are Pc targets based on data from Kwong et al. (2008) and Filion et al. (2010), and TRFs shaded light blue are Pc targets based on data from Kwong et al. (2008). See also Supplemental Table S2.

Hierarchical clustering of TRF binding across chromatin states reveals three primary chromatin-type preferences. The largest cluster consists of TRFs that primarily bind DNA in the YELLOW ‘active’ chromatin state. This cluster consists of many of the general factors described above as promoter-associated (HDACs, etc.) (Fig. 1A). However, a number of DNA-binding factors such as Ttk, Hr78, and Eip74EF also preferentially bind YELLOW chromatin. TRFs falling into the RED cluster are almost entirely DNA-binding factors including Trl (also known as GAGA factor, or GAF), a multifunctional regulator of gene expression, and a number of factors that drive tissue-specific patterns of expression, such as Pan, EcR, USP, as well as many of the mesodermal TRFs (Mef2, Twi, Bin, etc.) (Fig. 1A; Yao et al. 1992; Ciglar and Furlong 2009; Archbold et al. 2012).

There are three ‘inactive’ chromatin states according to the five-state model: BLACK, BLUE, and GREEN. GREEN chromatin is repressive heterochromatin, primarily pericentric, and is targeted by HP1 and little else (Fig. 1A). However, we observed that a large proportion of TRFs target BLACK and BLUE chromatin. BLACK chromatin covers approximately half of the genome and is relatively gene-poor; the genes that are associated with it are generally expressed in a tissue-specific manner. BLUE chromatin is Pc-targeted repressive chromatin. The Class II insulator protein Su(Hw) (Nègre et al. 2010) has a strong preference for BLACK chromatin across developmental contexts (early embryo, late embryo, and white prepupal stages). Many important regulators of early developmental patterning (Bcd, Ftz, H, Slp1, etc.) preferentially bind BLACK and BLUE chromatin to approximately the same degree (Fig. 1A; Levine and Davidson 2005; Sen et al. 2010). Finally, a set of developmental TRFs including Kni, Tll, Hth, Gro, Tin, Kr, and Cad (Sauer et al. 1996; Levine and Davidson 2005; Ciglar and Furlong 2009; Mann et al. 2009) preferentially binds BLUE chromatin over all other types (Fig. 1A).

**Figure 2.**
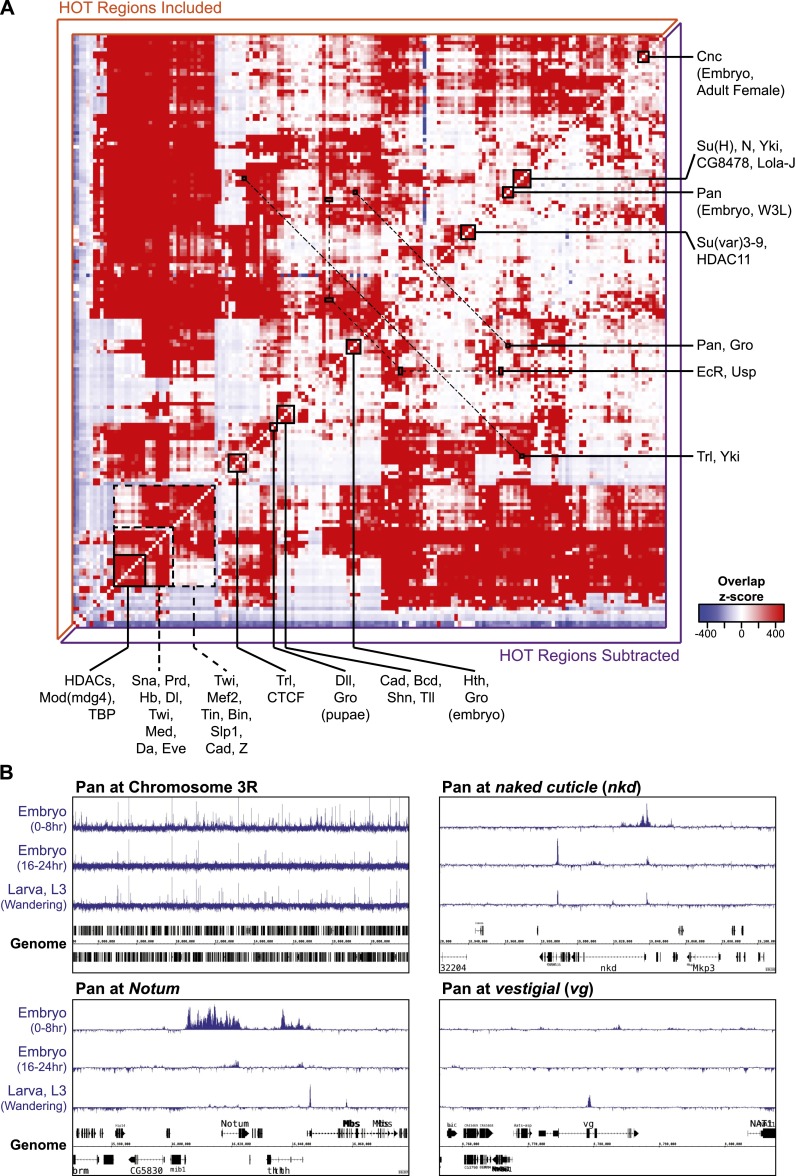
TRF–TRF colocalization matrix. (*A*) Significance of binding site overlap for pairwise TRF–TRF comparisons; shading represents *Z*-score enrichment (red) or depletion (blue). (*B*) Pan binding across chromosome 3R (∼14 Mb), and the *Notum*, *naked cuticle* (*nkd*), and *vestigial* (*vg*) loci at early embryonic, late embryonic, and larval developmental stages. See also Supplemental Table S3.

Interestingly, many of the TRFs that preferentially bind Pc-targeted DNA regions—BLUE chromatin—are involved in providing cell, tissue, or regional identities; these factors are often referred to as selector or selector-like genes in *Drosophila* (Garcia-Bellido 1975; Mann and Morata 2000). Because of their significant impact on cellular identity, the expression of selector-like genes must be precisely controlled. Therefore, we used the genome-wide ChIP data to ask whether TRFs that preferentially bind Pc-targeted DNA regions are Pc targets themselves. Indeed, of the 17 TRFs with >35% binding in BLUE chromatin, 14 (>80%) contain at least 100 bp of BLUE chromatin within their gene units (i.e., Pc targeted) (Fig. 1B). Further, two of the three exceptions have been identified as Pc targeted in another genome-wide study, and Pc indirectly regulates the lone factor that is not a direct Pc target (Stat92E) (see Discussion) (Kwong et al. 2008). These data indicate that many of the Pc-targeted, selector-like genes may fall within a partially self-contained Pc regulatory network.

### Coincident binding of transcriptional regulators

Precise spatiotemporal patterns of gene expression require the integration of multiple regulatory inputs from TRFs at enhancers. Interactions between transcriptional regulators at enhancers can be direct or indirect, and combined inputs can be additive, cooperative, or antagonistic (Slattery et al. 2011; Lelli et al. 2012; Spitz and Furlong 2012). To uncover potential coordinated regulatory interactions we calculated the genome-wide binding correlations for all transcriptional regulator pairs to identify TRFs with similar binding profiles. Many putative interactions are evident, and expected relationships are clear from the overlap matrix (Fig. 2A). For instance, EcR and USP are known to physically interact on regulatory enhancers, and these factors have similar binding profiles at multiple stages of development (Fig. 2A; Yao et al. 1992). Similarly, binding of the transcriptional corepressor Gro overlaps significantly with Pan, consistent with previous studies, and we also find clusters of cobinding for early embryo TRFs and mesoderm TRFs (Fig. 2A; Cavallo et al. 1998; Li et al. 2008; Spitz and Furlong 2012). The corepressor Gro also overlaps significantly with Hth and Dll, indicating that these factors may use Gro when regulating transcriptional repression, a possibility that has been suggested previously for Hth (Gebelein et al. 2004). In addition to these examples, a number of TRFs were tested at multiple stages of development (e.g., Trl, Pan, Cnc) and binding profiles from different stages tend to show significant overlap (Fig. 2A). That is, the global trend for these factors is similar across development. However, in these cases, there is still context-dependent binding variation at potentially significant loci. For example, Pan binding is quite consistent across multiple developmental stages, but varies significantly at loci such as *Notum*, *naked cuticle* (*nkd*), and *vestigial* (*vg*), all previously characterized Pan targets (Fig. 2B; Klein and Arias 1999; Schweizer et al. 2003; Fang et al. 2006; Chang et al. 2008).

Although many distinct, and expected, interactions are evident, the highly overlapping nature of TRF binding is also clear in the cobinding matrix. In fact, 5692 regions are bound by at least 14 factors (see Methods); in keeping with the previous literature, we refer to these hotspots of TRF localization as HOT (high-occupancy target) regions. Subtracting HOT regions from the calculation of TRF–TRF overlap significance leads to a more defined interaction matrix, often further highlighting the pairwise interactions described in the previous paragraph (Fig. 2A).

**Figure 3.**
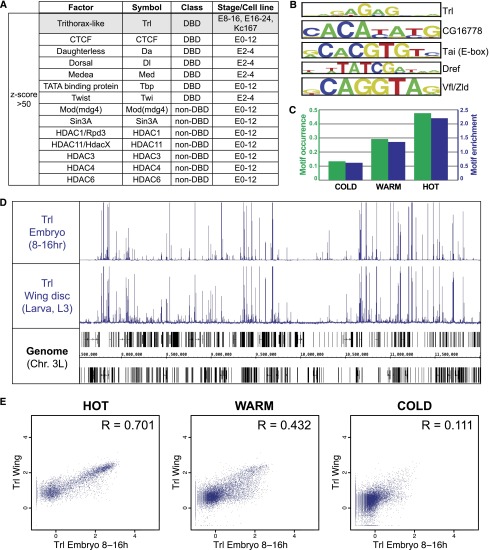
GAGA factor binding at HOT regions. (*A*) TRFs with data sets highly enriched for binding at HOT regions (*Z*-score >50). Only Trithorax-like/GAGA factor (Trl; shaded gray) is enriched at mid- and late-embryo stages and Kc167 cells. (*B*) Top five motifs enriched in HOT regions. Motif scanning was performed using FIMO (Grant et al. 2011). (*C*) Fraction of Trl consensus motif occurrence (green bars) and enrichment relative to random (blue bars) in COLD, WARM, and HOT regions. Trl consensus motif is as described in Adams et al. (2000). (*D*) Trl binding across chromosome 3L in embryos and dissected larval (L3) wing discs. (*E*) Scatterplots representing the Trl max ChIP-seq signal in embryo and wing disc data sets across HOT, WARM, and COLD regions; Pearson correlation (R) is indicated for each set of comparisons.

We further explored the extensive TRF colocalization by looking for the TRFs most enriched for binding in HOT regions. Seven DNA-binding factors and seven non-DNA-binding factors were very highly enriched in HOT regions (*Z*-score >50, see Methods) (Fig. 3A; Supplemental Table S4). With one exception the experiments for all of these enriched factors overlap early- to mid-embryogenesis; the outlier is Tfl, which is enriched for HOT region targeting during mid-embyogenesis (8–16 h), late-embryogenesis (16–24 h), and in Kc167 cells. An important role for Trl in the formation of HOT regions was suggested previously based on the overrepresentation of GAGA motifs—the Trl DNA-binding motif—across ∼2000 regions of high transcription-factor occupancy (Kvon et al. 2012). Indeed, we find that the GAGA motif is progressively enriched with increasing TRF occupancy (Fig. 3B,C; Supplemental Table S4). Interestingly, we find that Trl binding is remarkably consistent across development, with little variation between ChIPs performed with embryo chromatin (8–16 h) and ChIPs performed with chromatin from dissected larval wing discs (third instar) (Fig. 3D). Indeed, binding in HOT regions is driving this context-independent Trl binding (Fig. 3E). Thus, although the majority of our data are from embryonic stages, the lack of developmental variation in Trl binding to HOT regions suggests that HOT regions might also remain consistent throughout development.

### Low-, medium-, and high-occupancy target regions

**Figure 4.**
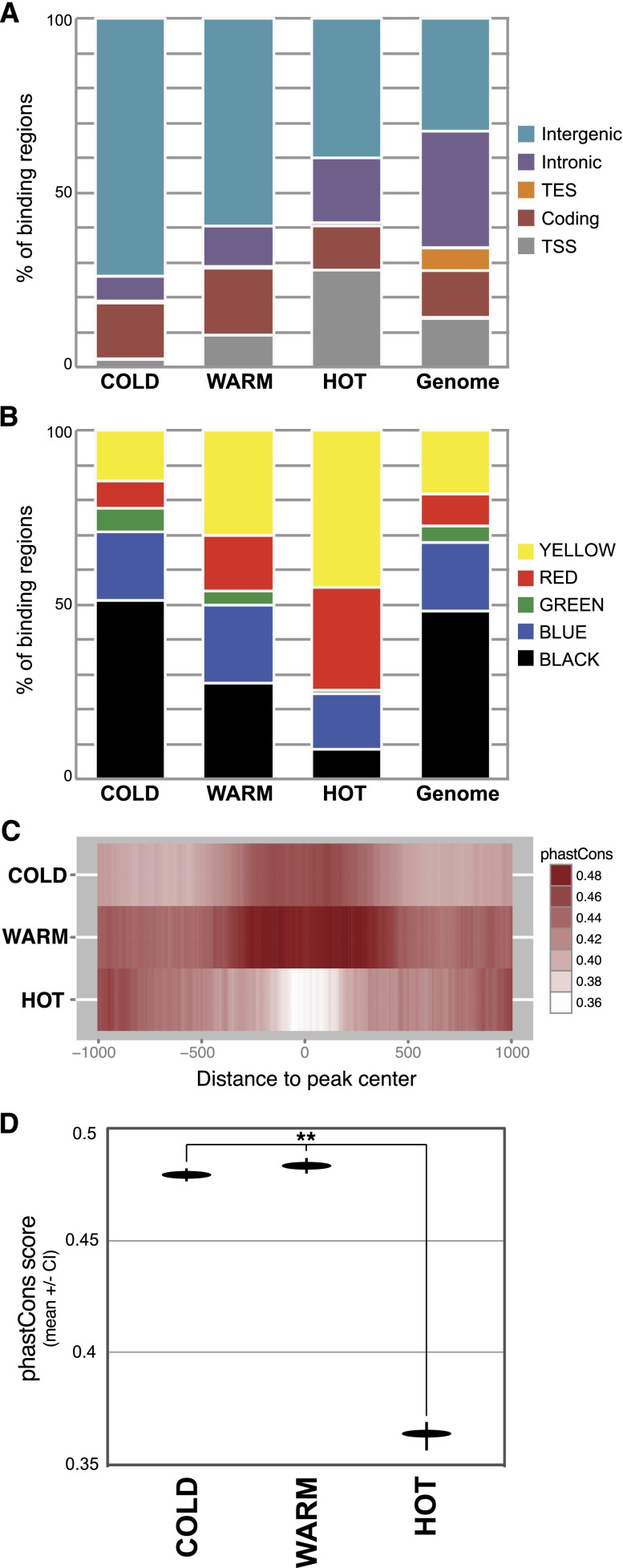
Characteristics of high- and low-occupancy binding regions. (*A*) Fraction of HOT, WARM, and COLD binding across the genomic regions described in Supplemental Figure S1. (*B*) Fraction of HOT, WARM, and COLD binding across the chromatin states described in Figure 1. (*C*) Mean phastCons score (10-bp window) across HOT, WARM, and COLD regions (center of region ±1 kb). (*D*) Mean phastCons score ± confidence interval (95%) for the central 100 bp of HOT, WARM, and COLD regions. Asterisks represent significant divergence from random ([**]*P* < 1 × 10^−15^; Wilcoxon rank sum test).

Aside from high TRF co-occupancy, do HOT regions have any other distinguishing features? To address this question we separated binding regions into three categories: HOT, WARM, and COLD; HOT regions are targeted by 14 or more TRFs, WARM regions are targeted by 4–13 TRFs, and COLD regions are targeted by 1–3 TRFs (see Methods). Relative to COLD regions, HOT regions occupy a much smaller fraction of the intergenic genome (Fig. 4A). The reverse trend is seen in regions around transcription start sites. In both cases, the patterns of WARM-binding regions fall in the intermediate range between HOT and COLD. The divergent patterns of HOT and COLD binding are also evident when looking at overlap with chromatin state: HOT regions are especially enriched for binding in RED and YELLOW (i.e., active) chromatin states, and depleted in all three inactive/repressed chromatin states, particularly BLACK and GREEN chromatin (Fig. 4B). Additionally, and consistent with previous reports, we found TRFs’ DNA motifs tend to be enriched binding sites that fall within COLD and/or WARM regions, and depleted in binding sites that fall within HOT regions (Supplemental Fig. S3).

We next asked whether there are differences in DNA conservation across the range of TRF occupancies. Our measure of DNA conservation in this case is its phastCons score, which measures the probability that an individual base is part of a stretch of base pairs (usually ∼100–1000 bp in length) that is conserved. One implication from such analysis is that DNA with a higher phastCons score is under purifying selection and more likely to be functional. Mean phastCons values were calculated for the center ±1 kb for all HOT, WARM, or COLD regions (Fig. 4C). Intriguingly, the patterns of conservation at HOT regions deviate from those at the lower occupancy regions. Generally speaking, the central 500 bp of COLD and WARM regions is more conserved than the central 500 bp of HOT regions (Fig. 4C). In addition, whereas the lower occupancy regions display a distinct pattern of increased conservation near their centers, the HOT regions actually have the opposite pattern, with less conservation near their centers relative to the distal edges. This pattern at HOT regions is due to their tendency to fall at promoter regions, with the increased distal conservation reflective of nearby coding regions. Focusing on the central 100 bp of HOT, WARM, and COLD regions reveals that HOT regions are significantly less conserved than WARM/COLD regions (Fig. 4D). Interestingly, promoter-proximal HOT regions drive this pattern, as promoter-distal HOT regions have a conservation pattern similar to WARM and COLD regions (Supplemental Fig. S4A). In fact, promoters associated with HOT regions are significantly less conserved than promoters that do not overlap HOT regions (Supplemental Fig. S4B). Thus, although many HOT regions are less evolutionarily constrained, it is clear that the promoter-distal HOT regions and non-HOT binding regions are centered on domains of increased DNA conservation, suggesting that these regions are likely to be functional.

**Figure 5.**
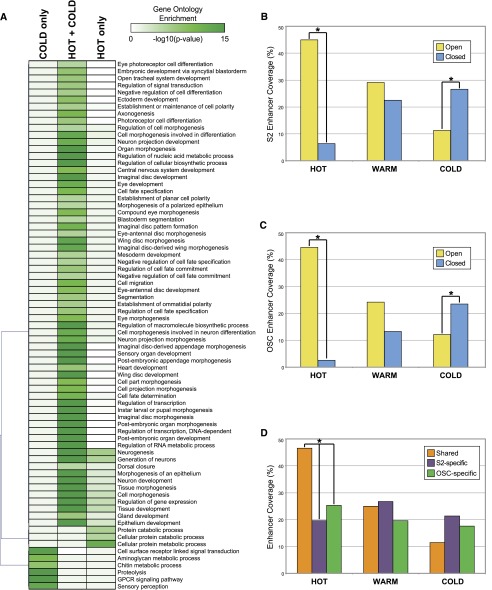
Enhancers targeted by high- and low-occupancy binding regions. (*A*) Heatmap representing gene ontology categories enriched across loci targeted by HOT regions only, COLD regions only, and loci targeted by both HOT and COLD regions. Shading represents −log_10_(*P*-value) (Bonferroni corrected). (*B*) Fraction of S2 cell STARR-seq enhancers that overlap HOT, WARM, and COLD regions. ‘Open’ and ‘closed’ enhancer categories are as characterized by Kvon et al. (2012). (*C*) Same as *B*, only for enhancers characterized in the OSC cell line. (*D*) Fraction of STARR-seq enhancers active in both S2 and OSC cells (Shared), enhancers active only in S2 cells (S2-specific), and enhancers active only in OSC cells (OSC-specific) that overlap with HOT, WARM, and COLD regions. For *B–D*, comparisons marked with an asterisk represent significant differences (*P* < 1 × 10^−20^, χ^2^ test).

With regard to target genes, HOT regions are often associated with genes that are highly and ubiquitously expressed housekeeping genes, consistent with the gene classes that fall within the YELLOW chromatin state. This is clear from gene ontology (GO) analysis of the genes associated with HOT regions: Categories such as ‘metabolic process’ and ‘cellular component biogenesis’ are highly significant (both *P* < 10^−15^). Additionally, non-housekeeping categories including ‘developmental process’ and ‘transcription regulator activity’ are also enriched (both *P* ≤ 10^−25^) among HOT target genes. However, comparing the gene sets targeted by HOT, WARM, and COLD regions becomes complicated because many loci are associated with more than one type of binding event. Thus, we instead focused on genes targeted by various combinations of HOT and COLD binding to explore potential differences in regulatory logic across unique gene sets. We broke genes down into loci associated only with a HOT binding region, loci associated with both HOT and COLD binding, and loci associated only with COLD binding. GO analysis of these gene categories revealed an interesting pattern for genes associated with HOT regions (Fig. 5A). Genes with HOT input but no COLD input are enriched for housekeeping categories (e.g., cellular protein metabolic process, cellular protein catabolic process). Genes receiving both HOT and COLD regulatory input, on the other hand, are enriched for categories associated with transcription and developmental patterning, morphogenesis, and differentiation (e.g., cell-fate specification, organ morphogenesis, neuron development, regulation of transcription). Together these results suggest that both ubiquitous housekeeping genes and highly regulated developmental genes are associated with regions of high TRF co-occupancy; developmental genes, however, require more specific TRF inputs as well, likely to provide the combinatorial regulatory inputs that are necessary to drive precise patterns of gene expression.

Multiple results described thus far are consistent with a model in which lower occupancy binding regions (COLD and WARM) represent the traditional developmentally regulated enhancer—these regions are conserved, often distal to the promoter, and tend to be associated with genes involved in developmental patterning. However, HOT regions can be associated with these patterning genes but are also associated with ubiquitous genes and tend to be promoter-proximal. It is important to point out that HOT regions are capable of driving patterned expression, as was recently demonstrated by Kvon et al. (2012) in a study of 108 HOT regions. However, the DNA regions tested in the aforementioned study tended to be near developmentally regulated genes and located in BLUE or RED, rather than YELLOW, chromatin (Supplemental Fig. S5). Thus, although this study unequivocally demonstrated that HOT regions are able to drive patterned gene expression, it may not be representative of HOT regions as a whole. For this reason, we chose to explore the relationship between TRF occupancy and regulatory activity on a global scale. We asked whether enhancers genome wide are more likely to be associated with high-, medium-, or low-occupancy target regions. To address this question, we compared the genome-wide binding data with a recently published genome-wide assessment of *Drosophila* enhancer activity, in which STARR–seq (self-transcribing active regulatory region sequencing) was used to identify two primary types of enhancers (Arnold et al. 2013). The first enhancer class, ‘open’ enhancers, overlaps accessible (DNase I hypersensitive) DNA regions; the second class of enhancers, ‘closed’ enhancers, does not overlap accessible DNA and appears to be epigenetically regulated at the chromatin level (Arnold et al. 2013). Overall, 2211 HOT regions overlap an enhancer, 2089 WARM regions overlap an enhancer, and 1617 COLD regions overlap an enhancer. A comparison of HOT, WARM, and COLD regions to the two classes of enhancers across S2 and OSC cells yielded interesting patterns of enrichment: in both cell types, HOT regions are significantly enriched for binding to open enhancers, whereas COLD regions are significantly enriched for binding to closed enhancers (Fig. 5B,C). Warm regions are enriched for binding both classes. Further, HOT regions are significantly more likely to occupy enhancers that are active across both cell types (Fig. 5D). Thus, all classes of binding are likely to overlap DNA regions with enhancer activity but, consistent with the chromatin state overlap, HOT regions are more likely to be associated with open enhancers used across cell types, whereas lower occupancy regions tend to be associated with complex enhancers that are also regulated at the epigenetic level.

## Discussion

In this study we provide analysis of new and previously published ChIP data, in combination with published chromatin state and genome-wide enhancer characterization data, to provide a window into the principles governing genomic gene regulatory networks and TRF–DNA interactions.

From a gene regulatory network perspective, this work highlights numerous interactions that may warrant further exploration. Thousands of TRF–DNA interactions are observed within these data, with 5823 binding events overlapping DNA regions that act as transcriptional enhancers (Arnold et al. 2013; Supplemental Table S5). A comparison of the TRF–DNA interactions across this diverse set of transcriptional regulators has identified numerous cobinding events that highlight direct or indirect interactions between TRFs on the same DNA regions. Multiple factors known to physically interact (EcR-USP, Pan-Gro, Yki-Trl) are identified as significantly colocalized on DNA across the genome (Yao et al. 1992; Cavallo et al. 1998; Oh et al. 2013), suggesting that additional colocalization relationships (e.g., Homothorax and Groucho) may represent functional interactions worth exploring in greater detail.

A broad range of binding strategies and preferences are clear from the TRFs analyzed in these data. Although a majority of the factors tend to bind proximal promoter regions, many important developmental regulators are significantly bound to intronic and promoter-distal intergenic regions, likely representing targeting of enhancers controlling genes subject to complex developmentally regulated transcriptional controls. TRFs also differ in their association with various types of chromatin. Most TRFs preferentially bind one of the two ‘active’ chromatin states (RED, YELLOW), consistent with the overall accessibility of these chromatin types and their association with expressed genes.

Not all TRFs bind primarily in the active chromatin states, however. For example, a number of TRFs (Fig. 1B) are often bound to genomic regions that fall into the BLUE chromatin state. BLUE chromatin is a Pc-targeted chromatin and is associated with the H3K27me3 repressive histone modification (Simon and Kingston 2009; Filion et al. 2010; van Steensel 2011). Interestingly, the factors that favor Pc-targeted chromatin tend to be Pc targets themselves. Indeed, there are 20 data sets, representing 17 TRFs, in which >35% of binding events overlap BLUE/Pc chromatin; 14 of these TRFs are also targeted by Pc as evidenced by the fact that their genic regions overlap BLUE chromatin (and based on published data). The three exceptions are Pcl, Tll, and Stat92E. Pcl (Polycomb-like) is a Polycomb group protein that has been identified as Pc targeted in another genome-wide study that covered multiple stages of development (Kwong et al. 2008; Filion et al. 2010). Tll has been previously characterized as a Pc target, though Pc binding is developmentally transient and was likely missed by the Kc cell-based chromatin state classifiers for this reason (Kwong et al. 2008). And Stat92E is the terminal transcription factor of the JAK/STAT signaling pathway (Yan et al. 1996). Although Stat92E does not appear to be regulated by Pc, the three genes encoding the Unpaired (Upd) family of JAK/STAT pathway ligands—*os* (*outstretched*, also known as *unpaired*), *upd2*, and *upd3*—fall within a large domain of BLUE chromatin and are confirmed Pc targets (Classen et al. 2009). Ligand-mediated activation of JAK/STAT by Upd is the rate-limiting step dictating Stat92E activity, so this is another case in which a transcription factor’s activity is regulated by Pc (Classen et al. 2009). Thus, all of the factors that significantly bind to Pc-targeted DNA regions are in turn regulated by Pc. This strategy, in which both TRFs and their targets are subject to heritable epigenetic control, highlights a strict multi-tiered mechanism that can be used to ensure precise and reproducible development of a multicellular organism. The work presented here, although it does not exhaust the repertoire of Pc-regulated genes, further generalizes this view and underscores the importance of PcG regulation for ensuring the somatically heritable, high-fidelity maintenance of the spatially restricted patterns of expression of such developmentally important transcriptional regulators.

Exploration of the patterns of TRF occupancy across the genome revealed thousands of regions bound by >14 TRFs. It has previously been shown that the *D. melanogaster*, *Caenorhabditis elegans*, and human genomes all contain regions of DNA that are bound by numerous, often unrelated, transcriptional regulators (Moorman et al. 2006; Nègre et al. 2011; Niu et al. 2011; Yip et al. 2012). These “HOT” regions often do not contain the expected DNA motifs for the bound TRFs and binding may be mediated in part via protein–protein interactions (Moorman et al. 2006; Kvon et al. 2012). Although this widespread, possibly indirect, binding has led to the suggestion that HOT regions might drive ubiquitous expression, in *Drosophila* HOT regions can drive patterned gene expression (Kvon et al. 2012). Thus, in terms of *cis*-regulatory activity, HOT regions are sometimes similar to traditional enhancers. Nevertheless, we have identified multiple properties of HOT regions that distinguish them from lower-occupancy TRF-binding regions. Relative to lower-occupancy target regions, HOT regions are more likely to occur in proximal promoter regions, and more likely to fall in YELLOW and RED chromatin regions, and more likely to be associated with highly expressed housekeeping genes. Overall, this indicates HOT regions are generally associated with the highly accessible DNA found at the promoters of housekeeping genes. However, a subset of HOT regions is associated with developmental genes, and these genes often have additional non-HOT regulatory inputs.

Consistent with the overrepresentation of GAGA motifs in HOT regions (Fig. 3; Kvon et al. 2012), we find binding of Trl to be highly enriched in HOT regions across multiple developmental contexts, suggesting that Trl may play an important role in maintaining HOT regions or influencing the regulatory output of HOT regions. Trl plays a role in directing nucleosome turnover and is associated with regions of low-nucleosome occupancy (Petesch and Lis 2008; Deal et al. 2010; Li and Gilmour 2013) and interacts with the FACT and NURF chromatin remodeling complexes (Xiao et al. 2001; Shimojima et al. 2003), putting it in a position to maintain the accessibility of HOT regions. Additionally, the GAGA motif has been associated with paused RNA polymerase II (Pol II), and Trl has recently been shown to recruit NELF (negative elongation factor) to promoters, putting it in a position to modulate the release of paused Pol II (Hendrix et al. 2008; Lee et al. 2008; Gilchrist et al. 2010; Fay et al. 2011; Li et al. 2013). Thus, through interaction with NELF, Trl is also in a position to directly regulate gene expression at promoter-proximal HOT regions.

Surprisingly, HOT regions are generally less evolutionarily constrained than lower-occupancy TRF-binding regions. Across their central 100 bp, COLD and WARM regions are significantly more conserved than HOT regions. There are at least two possibilities that could explain this finding. One possibility is that HOT regions have a locally elevated mutation rate. Indeed, we see a pattern similar to the phastCons pattern when looking at SNP density across *D. melanogaster* populations: SNP density in HOT regions is significantly higher than in lower-occupancy regions (Supplemental Fig. S6). Consistent with the promoter bias of HOT regions, some evidence suggests that SNP density increases in the proximal promoter in *D. melanogaster* and humans and, additionally, SNPs in the proximal promoter show a biased signature of transversions in humans (Guo and Jamison 2005; Main et al. 2013). Perhaps the accessibility of DNA in promoter-proximal HOT regions leads to increased exposure to insults that cause mutation. Alternatively, much of the binding at HOT regions appears to be functionally neutral and possibly indirect (Moorman et al. 2006; Kvon et al. 2012); thus, the mode of binding at HOT regions may allow for rapid DNA sequence turnover or insertions/deletions as long as accessibility is maintained. Conversely, DNA motif sequence and the spacing between motifs are functionally constrained at many, but not all, *cis*-regulatory modules, and this too is the case with lower-occupancy TRF-binding regions (Erives and Levine 2004; Arnosti and Kulkarni 2005; Borok et al. 2010; Swanson et al. 2010; Evans et al. 2012). This, combined with the fact that WARM and COLD regions are more likely to fall distal to the promoter, suggests that lower-occupancy TRF regions often represent traditional enhancers.

Despite their differences, however, HOT, WARM, and COLD regions are all significantly enriched for binding DNA with enhancer activity. But once again the evidence suggests that the type of enhancers targeted differ across the occupancy groups. HOT regions are more likely to occur in highly accessible ‘open’ enhancers that direct gene expression in a context-independent fashion (at least across S2 and OSC cell lines), whereas lower-occupancy regions are more likely to target less accessible enhancers that tend to be further regulated at the epigenetic level. In combination with the results described above, this provides another piece of evidence that lower-occupancy regions represent traditional enhancers, which tend to be subject to more complex spatial and developmental regulation, while HOT regions represent DNA regions with context-independent, and possibly less complex, regulatory functions.

## Methods

### Chromatin immunoprecipitation

Chromatin collection and chromatin immunoprecipitation were performed as described previously (Nègre et al. 2011). Transgenic lines containing GFP-tagged transcription factors within their endogenous genomic contexts were produced using the P[acman] bacterial artificial chromosome (BAC) system as previously described (Venken et al. 2009; Roy et al. 2010). Antibody details are available at modMine (http://intermine.modencode.org). A number of antibodies were generous contributions from members of the *Drosophila* research community: Ken Cadigan (Pan/TCF), Andy Dingwall (Cmi/Lpt), Eric Lai (Insv), Erika Bach (Stat92E), Jim Kadonaga (TBP), Ken Irvine (Yki), Claude Desplan (Prd), Stephen Crews (Sc), Sean Carroll (Dll), Richard Mann (Hth), and Scott Hawley (Trem). Immunoprecipitated DNA was prepared for Illumina sequencing either as described in Nègre et al. (2011) or using the Epicentre Nextera DNA Sample Preparation Kit. Briefly, Nextera library preparations were performed using the High Molecular Weight tagmentation buffer, and tagmented DNA was amplified using 12 cycles of PCR. DNA was then sequenced on an Illumina HiSeq 2000 according to the manufacturer’s standard protocols.

### Data processing

ChIP-chip peak calls are as previously described (Li et al. 2008; MacArthur et al. 2009; Zinzen et al. 2009; Nègre et al. 2011). For ChIP-seq experiments, biological replicates were scored against an appropriate input DNA control (from non-immunoprecipitated chromatin). The MACS (v2) peak caller was used to identify and score (rank) potential binding sites/peaks (Zhang et al. 2008). For obtaining optimal thresholds, we used the irreproducible discovery rate (IDR) framework to determine high-confidence binding events by leveraging the reproducibility and rank consistency of peak identifications across replicate experiments of a data set (Li et al. 2011a). Briefly, for individual replicates, peaks were called using MACS2 with a *P*-value threshold of 1 × 10^−3^ to obtain a maximum of 30-k peaks, and peaks were ranked according to their *P*-value scores. Replicates were then pooled and MACS2 was again used to call peaks at a *P*-value threshold of 1 × 10^−3^. Peaks from the pooled set that overlapped at least one peak in both individual replicate sets were retained. From this set of replicate-reproducible peaks, we obtained two independent rankings based on the *P*-values from each replicate; this pair of ranked lists was used as input for the IDR framework. Cross-replicate and pseudoreplicate rank thresholds at an IDR of 5% were generated, and the better of the two was used to generate the final set of rank consistent and reproducible peaks. Details of the IDR framework are available at https://sites.google.com/site/anshulkundaje/projects/idr. All data sets are available through the modENCODE Data Coordinating Center (DCC; see Supplemental Table S1 for DCC submission IDs).

### Peak annotation and TRF-binding overlap

ChIP peaks were annotated as overlapping genomic features according to the FlyBase r5.34 gene structure annotation and the following categories: promoter (transcription start site; within 1-kb upstream of a transcription start site or overlapping 5′ UTR), coding (coding sequence), downstream (transcription stop site; 3′ UTR or 200-bp downstream from gene), intron, intergenic regions. Peaks were assigned to target genes based on the nearest transcription start site (≤10,000 bp). Peaks were assigned to chromatin states based on their overlap with the five states defined in Filion et al. (2010), using the priority order of BLUE>RED>YELLOW>GREEN>BLACK in cases where one peak overlapped multiple chromatin types.

To calculate the significance of cobinding for two TRFs, the occurrence of colocalization of each pair of ChIP peak sets was compared with a permutated background performed 10,000 times, and a *Z*-score was assigned to each pair to indicate whether the co-occurrence was significantly higher or lower than expectation (see http://www.encodestatistics.org/). Regions of significant cobinding—HOT regions—were defined following the algorithm described in Nègre et al. (2011). Briefly, to establish HOT regions based on the colocalization of all 171 regulators on the *D. melanogaster* genome, we took centers of all peaks of all regulators to represent their genomic coordinates and calculated the density genome widely using 300-bp bandwidth Kernel Density Estimation. We then scanned the density scores peak wide and denoted each peak a HOT region candidate. The complexity (occupancy) of each HOT region candidate was calculated by summing the Gaussian kernalized distance from the peak to peaks of each other regulator that contributed at least 0.1 to this strength. Finally, we named these candidate regions as HOT if the complexity was ≥15, as COLD if the complexity was ≤3, and as WARM for all the rest. HOT, WARM, and COLD regions were assigned to genomic features, target genes, and chromatin states as described above for individual TRFs. Only level 5 Gene Ontology (GO) categories in which the enrichment *P*-value (Bonferroni corrected) was ≤1 × 10^−5^ for at least one of the three categories (COLD only; HOT + COLD; HOT only) were used for GO category clustering (Fig. 5). The top motifs enriched in HOT regions (Fig. 5) were identified using FIMO (Grant et al. 2011).

## Data access

Data from this study have been submitted to the NCBI Gene Expression Omnibus (GEO; http://www.ncbi.nlm.nih.gov/geo/), modMine (http://intermine.modencode.org), or both under GEO accession nos. GSE49768–GSE49780, GSE49899, or the modMINE DCC IDs 2627, 2629, 2630, 2633, 2634, 2636, 3234, 3240, 3390–3396, 3399–3401, 3403. See Supplemental Table S1 for all data set submission information.

## References

[B1] Adams MD, Celniker SE, Holt RA, Evans CA, Gocayne JD, Amanatides PG, Scherer SE, Li PW, Hoskins RA, Galle RF, 2000 The genome sequence of *Drosophila melanogaster*. Science 287: 2185–2195 10731132 10.1126/science.287.5461.2185

[B2] Andrew DJ, Scott MP 1994 Immunological methods for mapping protein distributions on polytene chromosomes. Methods Cell Biol 44: 353–370 7707963 10.1016/s0091-679x(08)60923-1

[B3] Archbold HC, Yang YX, Chen L, Cadigan KM 2012 How do they do Wnt they do?: regulation of transcription by the Wnt/β-catenin pathway. Acta Physiol 204: 74–109 10.1111/j.1748-1716.2011.02293.x 21624092

[B4] Arnold CD, Gerlach D, Stelzer C, Boryn LM, Rath M, Stark A 2013 Genome-wide quantitative enhancer activity maps identified by STARR-seq. Science 339: 1074–1077 23328393 10.1126/science.1232542

[B5] Arnosti DN, Kulkarni MM 2005 Transcriptional enhancers: intelligent enhanceosomes or flexible billboards? J Cell Biochem 94: 890–898 15696541 10.1002/jcb.20352

[B6] Ashburner M 1967 Gene activity dependent on chromosome synapsis in the polytene chromosomes of *Drosophila melanogaster*. Nature 214: 1159–1160 6053090 10.1038/2141159b0

[B7] Ashburner M 1970 The genetic analysis of puffing in polytene chromosomes of *Drosophila*. Proc Royal Soc London B Biol Sci 176: 319–327 10.1098/rspb.1970.0052 4395102

[B8] Biggin MD 2011 Animal transcription networks as highly connected, quantitative continua. Dev Cell 21: 611–626 22014521 10.1016/j.devcel.2011.09.008

[B9] Borok MJ, Tran DA, Ho MC, Drewell RA 2010 Dissecting the regulatory switches of development: lessons from enhancer evolution in *Drosophila*. Development 137: 5–13 20023155 10.1242/dev.036160 PMC2796927

[B10] Cavallo RA, Cox RT, Moline MM, Roose J, Polevoy GA, Clevers H, Peifer M, Bejsovec A 1998 *Drosophila* Tcf and Groucho interact to repress Wingless signalling activity. Nature 395: 604–608 9783586 10.1038/26982

[B11] Chang MV, Chang JL, Gangopadhyay A, Shearer A, Cadigan KM 2008 Activation of wingless targets requires bipartite recognition of DNA by TCF. Curr Biol 18: 1877–1881 19062282 10.1016/j.cub.2008.10.047 PMC3105462

[B12] Ciglar L, Furlong EE 2009 Conservation and divergence in developmental networks: a view from *Drosophila* myogenesis. Curr Opin Cell Biol 21: 754–760 19896355 10.1016/j.ceb.2009.10.001

[B13] Classen AK, Bunker BD, Harvey KF, Vaccari T, Bilder D 2009 A tumor suppressor activity of *Drosophila* Polycomb genes mediated by JAK-STAT signaling. Nat Genet 41: 1150–1155 19749759 10.1038/ng.445 PMC2782793

[B14] Deal RB, Henikoff JG, Henikoff S 2010 Genome-wide kinetics of nucleosome turnover determined by metabolic labeling of histones. Science 328: 1161–1164 20508129 10.1126/science.1186777 PMC2879085

[B15] Delest A, Sexton T, Cavalli G 2012 Polycomb: a paradigm for genome organization from one to three dimensions. Curr Opin Cell Biol 24: 405–414 22336329 10.1016/j.ceb.2012.01.008

[B16] Erives A, Levine M 2004 Coordinate enhancers share common organizational features in the *Drosophila* genome. Proc Natl Acad Sci 101: 3851–3856 15026577 10.1073/pnas.0400611101 PMC374333

[B17] Evans NC, Swanson CI, Barolo S 2012 Sparkling insights into enhancer structure, function, and evolution. Curr Top Dev Biol 98: 97–120 22305160 10.1016/B978-0-12-386499-4.00004-5

[B18] Fang M, Li J, Blauwkamp T, Bhambhani C, Campbell N, Cadigan KM 2006 C-terminal-binding protein directly activates and represses Wnt transcriptional targets in *Drosophila*. EMBO J 25: 2735–2745 16710294 10.1038/sj.emboj.7601153 PMC1500853

[B19] Fay A, Misulovin Z, Li J, Schaaf CA, Gause M, Gilmour DS, Dorsett D 2011 Cohesin selectively binds and regulates genes with paused RNA polymerase. Curr Biol 21: 1624–1634 21962715 10.1016/j.cub.2011.08.036 PMC3193539

[B20] Filion GJ, van Bemmel JG, Braunschweig U, Talhout W, Kind J, Ward LD, Brugman W, de Castro IJ, Kerkhoven RM, Bussemaker HJ, 2010 Systematic protein location mapping reveals five principal chromatin types in *Drosophila* cells. Cell 143: 212–224 20888037 10.1016/j.cell.2010.09.009 PMC3119929

[B21] Garcia-Bellido A 1975 Genetic control of wing disc development in *Drosophila*. Ciba Found Symp 0: 161–182 1039909 10.1002/9780470720110.ch8

[B22] Gebelein B, McKay DJ, Mann RS 2004 Direct integration of Hox and segmentation gene inputs during *Drosophila* development. Nature 431: 653–659 15470419 10.1038/nature02946

[B23] Gilchrist DA, Dos Santos G, Fargo DC, Xie B, Gao Y, Li L, Adelman K 2010 Pausing of RNA polymerase II disrupts DNA-specified nucleosome organization to enable precise gene regulation. Cell 143: 540–551 21074046 10.1016/j.cell.2010.10.004 PMC2991113

[B24] Grant CE, Bailey TL, Noble WS 2011 FIMO: scanning for occurrences of a given motif. Bioinformatics 27: 1017–1018 21330290 10.1093/bioinformatics/btr064 PMC3065696

[B25] Guertin MJ, Lis JT 2010 Chromatin landscape dictates HSF binding to target DNA elements. PLoS Genet 6 e1001114 20844575 10.1371/journal.pgen.1001114 PMC2936546

[B26] Guo Y, Jamison DC 2005 The distribution of SNPs in human gene regulatory regions. BMC Genomics 6: 140 16209714 10.1186/1471-2164-6-140 PMC1260019

[B27] Harding K, Rushlow C, Doyle HJ, Hoey T, Levine M 1986 Cross-regulatory interactions among pair-rule genes in *Drosophila*. Science 233: 953–959 3755551 10.1126/science.3755551

[B28] Hendrix DA, Hong JW, Zeitlinger J, Rokhsar DS, Levine MS 2008 Promoter elements associated with RNA Pol II stalling in the *Drosophila* embryo. Proc Natl Acad Sci 105: 7762–7767 18505835 10.1073/pnas.0802406105 PMC2396556

[B29] Jamrich M, Haars R, Wulf E, Bautz FA 1977 Correlation of RNA polymerase B and transcriptional activity in the chromosomes of *Drosophila melanogaster*. Chromosoma 64: 319–326 413700 10.1007/BF00294939

[B30] Kharchenko PV, Alekseyenko AA, Schwartz YB, Minoda A, Riddle NC, Ernst J, Sabo PJ, Larschan E, Gorchakov AA, Gu T, 2011 Comprehensive analysis of the chromatin landscape in *Drosophila melanogaster*. Nature 471: 480–485 21179089 10.1038/nature09725 PMC3109908

[B31] Klein T, Arias AM 1999 The vestigial gene product provides a molecular context for the interpretation of signals during the development of the wing in *Drosophila*. Development 126: 913–925 9927593 10.1242/dev.126.5.913

[B32] Kvon EZ, Stampfel G, Yanez-Cuna JO, Dickson BJ, Stark A 2012 HOT regions function as patterned developmental enhancers and have a distinct *cis*-regulatory signature. Genes Dev 26: 908–913 22499593 10.1101/gad.188052.112 PMC3347788

[B33] Kwong C, Adryan B, Bell I, Meadows L, Russell S, Manak JR, White R 2008 Stability and dynamics of polycomb target sites in *Drosophila* development. PLoS Genet 4: e1000178 18773083 10.1371/journal.pgen.1000178 PMC2525605

[B34] Lee C, Li X, Hechmer A, Eisen M, Biggin MD, Venters BJ, Jiang C, Li J, Pugh BF, Gilmour DS 2008 NELF and GAGA factor are linked to promoter-proximal pausing at many genes in *Drosophila*. Mol Cell Biol 28: 3290–3300 18332113 10.1128/MCB.02224-07 PMC2423147

[B35] Lelli KM, Slattery M, Mann RS 2012 Disentangling the many layers of eukaryotic transcriptional regulation. Annu Rev Genet 46: 43–68 22934649 10.1146/annurev-genet-110711-155437 PMC4295906

[B36] Levine M, Davidson EH 2005 Gene regulatory networks for development. Proc Natl Acad Sci 102: 4936–4942 15788537 10.1073/pnas.0408031102 PMC555974

[B37] Li J, Gilmour DS 2013 Distinct mechanisms of transcriptional pausing orchestrated by GAGA factor and M1BP, a novel transcription factor. EMBO J 32: 1829–1841 23708796 10.1038/emboj.2013.111 PMC3981175

[B38] Li XY, MacArthur S, Bourgon R, Nix D, Pollard DA, Iyer VN, Hechmer A, Simirenko L, Stapleton M, Luengo Hendriks CL, 2008 Transcription factors bind thousands of active and inactive regions in the *Drosophila* blastoderm. PLoS Biol 6: e27 18271625 10.1371/journal.pbio.0060027 PMC2235902

[B39] Li Q, Brown JB, Huang H, Bickel PJ 2011a Measuring reproducibility of high-throughput experiments. Ann Appl Stat 5: 1752–1779

[B40] Li XY, Thomas S, Sabo PJ, Eisen MB, Stamatoyannopoulos JA, Biggin MD 2011b The role of chromatin accessibility in directing the widespread, overlapping patterns of *Drosophila* transcription factor binding. Genome Biol 12: R34 21473766 10.1186/gb-2011-12-4-r34 PMC3218860

[B41] Li J, Liu Y, Rhee HS, Ghosh SK, Bai L, Pugh BF, Gilmour DS 2013 Kinetic competition between elongation rate and binding of NELF controls promoter-proximal pausing. Mol Cell 50: 711–722 23746353 10.1016/j.molcel.2013.05.016 PMC3695833

[B42] MacArthur S, Li XY, Li J, Brown JB, Chu HC, Zeng L, Grondona BP, Hechmer A, Simirenko L, Keranen SV, 2009 Developmental roles of 21 *Drosophila* transcription factors are determined by quantitative differences in binding to an overlapping set of thousands of genomic regions. Genome Biol 10: R80 19627575 10.1186/gb-2009-10-7-r80 PMC2728534

[B43] Main BJ, Smith AD, Jang H, Nuzhdin SV 2013 Transcription start site evolution in *Drosophila*. Mol Biol Evol 30: 1966–1974 23649539 10.1093/molbev/mst085 PMC3708499

[B44] Mann RS, Morata G 2000 The developmental and molecular biology of genes that subdivide the body of *Drosophila*. Annu Rev Cell Dev Biol 16: 243–271 11031237 10.1146/annurev.cellbio.16.1.243

[B45] Mann RS, Lelli KM, Joshi R 2009 Hox specificity unique roles for cofactors and collaborators. Curr Top Dev Biol 88: 63–101 19651302 10.1016/S0070-2153(09)88003-4 PMC2810641

[B46] Moorman C, Sun LV, Wang J, de Wit E, Talhout W, Ward LD, Greil F, Lu XJ, White KP, Bussemaker HJ, 2006 Hotspots of transcription factor colocalization in the genome of *Drosophila melanogaster*. Proc Natl Acad Sci 103: 12027–12032 16880385 10.1073/pnas.0605003103 PMC1567692

[B47] Nègre N, Hennetin J, Sun LV, Lavrov S, Bellis M, White KP, Cavalli G 2006 Chromosomal distribution of PcG proteins during *Drosophila* development. PLoS Biol 4: e170 16613483 10.1371/journal.pbio.0040170 PMC1440717

[B48] Nègre N, Brown CD, Shah PK, Kheradpour P, Morrison CA, Henikoff JG, Feng X, Ahmad K, Russell S, White RA, 2010 A comprehensive map of insulator elements for the *Drosophila* genome. PLoS Genet 6: e1000814 20084099 10.1371/journal.pgen.1000814 PMC2797089

[B49] Nègre N, Brown CD, Ma L, Bristow CA, Miller SW, Wagner U, Kheradpour P, Eaton ML, Loriaux P, Sealfon R, 2011 A *cis*-regulatory map of the *Drosophila* genome. Nature 471: 527–531 21430782 10.1038/nature09990 PMC3179250

[B50] Niu W, Lu ZJ, Zhong M, Sarov M, Murray JI, Brdlik CM, Janette J, Chen C, Alves P, Preston E, 2011 Diverse transcription factor binding features revealed by genome-wide ChIP-seq in *C. elegans*. Genome Res 21: 245–254 21177963 10.1101/gr.114587.110 PMC3032928

[B51] Oh H, Slattery M, Ma L, Crofts A, White KP, Mann RS, Irvine KD 2013 Genome-wide association of yorkie with chromatin and chromatin-remodeling complexes. Cell Reports 3: 309–318 23395637 10.1016/j.celrep.2013.01.008 PMC3633442

[B52] Ong CT, Corces VG 2012 Enhancers: emerging roles in cell fate specification. EMBO Rep 13: 423–430 22491032 10.1038/embor.2012.52 PMC3343362

[B53] Petesch SJ, Lis JT 2008 Rapid, transcription-independent loss of nucleosomes over a large chromatin domain at *Hsp70* loci. Cell 134: 74–84 18614012 10.1016/j.cell.2008.05.029 PMC2527511

[B54] Ritossa FM 1964 Experimental activation of specific loci in polytene chromosomes of *Drosophila*. Exp Cell Res 35: 601–607 14208747 10.1016/0014-4827(64)90147-8

[B55] Roy S, Ernst J, Kharchenko PV, Kheradpour P, Nègre N, Eaton ML, Landolin JM, Bristow CA, Ma L, Lin MF, 2010 Identification of functional elements and regulatory circuits by *Drosophila* modENCODE. Science 330: 1787–1797 21177974 10.1126/science.1198374 PMC3192495

[B56] Sauer F, Rivera-Pomar R, Hoch M, Jackle H 1996 Gene regulation in the *Drosophila* embryo. Philos Trans R Soc Lond B Biol Sci 351: 579–587 8735281 10.1098/rstb.1996.0057

[B57] Schweizer L, Nellen D, Basler K 2003 Requirement for Pangolin/dTCF in *Drosophila* Wingless signaling. Proc Natl Acad Sci 100: 5846–5851 12730381 10.1073/pnas.1037533100 PMC156289

[B58] Sen A, Stultz BG, Lee H, Hursh DA 2010 Odd paired transcriptional activation of decapentaplegic in the *Drosophila* eye/antennal disc is cell autonomous but indirect. Dev Biol 343: 167–177 20403347 10.1016/j.ydbio.2010.04.003

[B59] Sexton T, Yaffe E, Kenigsberg E, Bantignies F, Leblanc B, Hoichman M, Parrinello H, Tanay A, Cavalli G 2012 Three-dimensional folding and functional organization principles of the *Drosophila* genome. Cell 148: 458–472 22265598 10.1016/j.cell.2012.01.010

[B60] Shimojima T, Okada M, Nakayama T, Ueda H, Okawa K, Iwamatsu A, Handa H, Hirose S 2003 *Drosophila* FACT contributes to Hox gene expression through physical and functional interactions with GAGA factor. Genes Dev 17: 1605–1616 12815073 10.1101/gad.1086803 PMC196133

[B61] Silver LM, Elgin SC 1976 A method for determination of the in situ distribution of chromosomal proteins. Proc Natl Acad Sci 73: 423–427 813226 10.1073/pnas.73.2.423 PMC335921

[B62] Simon JA, Kingston RE 2009 Mechanisms of polycomb gene silencing: knowns and unknowns. Nat Rev Mol Cell Biol 10: 697–708 19738629 10.1038/nrm2763

[B63] Slattery M, Riley T, Liu P, Abe N, Gomez-Alcala P, Dror I, Zhou T, Rohs R, Honig B, Bussemaker HJ, 2011 Cofactor binding evokes latent differences in DNA binding specificity between Hox proteins. Cell 147: 1270–1282 22153072 10.1016/j.cell.2011.10.053 PMC3319069

[B64] Slattery M, Nègre N, White KP 2012 Interpreting the regulatory genome: the genomics of transcription factor function in *Drosophila melanogaster*. Brief Funct Genomics 11: 336–346 23023663 10.1093/bfgp/els034 PMC3459015

[B65] Spitz F, Furlong EE 2012 Transcription factors: from enhancer binding to developmental control. Nat Rev Genet 13: 613–626 22868264 10.1038/nrg3207

[B66] Swanson CI, Evans NC, Barolo S 2010 Structural rules and complex regulatory circuitry constrain expression of a Notch- and EGFR-regulated eye enhancer. Dev Cell 18: 359–370 20230745 10.1016/j.devcel.2009.12.026 PMC2847355

[B67] van Steensel B 2011 Chromatin: constructing the big picture. EMBO J 30: 1885–1895 21527910 10.1038/emboj.2011.135 PMC3098493

[B68] Venken KJ, Carlson JW, Schulze KL, Pan H, He Y, Spokony R, Wan KH, Koriabine M, de Jong PJ, White KP, 2009 Versatile P[acman] BAC libraries for transgenesis studies in *Drosophila melanogaster*. Nat Methods 6: 431–434 19465919 10.1038/nmeth.1331 PMC2784134

[B69] White R 2012 Packaging the fly genome: domains and dynamics. Brief Funct Genomics 11: 347–355 22945596 10.1093/bfgp/els020

[B70] Xiao H, Sandaltzopoulos R, Wang HM, Hamiche A, Ranallo R, Lee KM, Fu D, Wu C 2001 Dual functions of largest NURF subunit NURF301 in nucleosome sliding and transcription factor interactions. Mol Cell 8: 531–543 11583616 10.1016/s1097-2765(01)00345-8

[B71] Yan R, Small S, Desplan C, Dearolf CR, Darnell JE Jr 1996 Identification of a Stat gene that functions in *Drosophila* development. Cell 84: 421–430 8608596 10.1016/s0092-8674(00)81287-8

[B72] Yao TP, Segraves WA, Oro AE, McKeown M, Evans RM 1992 *Drosophila* ultraspiracle modulates ecdysone receptor function via heterodimer formation. Cell 71: 63–72 1327536 10.1016/0092-8674(92)90266-f

[B73] Yip KY, Cheng C, Bhardwaj N, Brown JB, Leng J, Kundaje A, Rozowsky J, Birney E, Bickel P, Snyder M, 2012 Classification of human genomic regions based on experimentally determined binding sites of more than 100 transcription-related factors. Genome Biol 13: R48 22950945 10.1186/gb-2012-13-9-r48 PMC3491392

[B74] Zeitlinger J, Stark A, Kellis M, Hong JW, Nechaev S, Adelman K, Levine M, Young RA 2007 RNA polymerase stalling at developmental control genes in the *Drosophila melanogaster* embryo. Nat Genet 39: 1512–1516 17994019 10.1038/ng.2007.26 PMC2824921

[B75] Zhang Y, Liu T, Meyer CA, Eeckhoute J, Johnson DS, Bernstein BE, Nusbaum C, Myers RM, Brown M, Li W, 2008 Model-based analysis of ChIP-Seq (MACS). Genome Biol 9: R137 18798982 10.1186/gb-2008-9-9-r137 PMC2592715

[B76] Zinzen RP, Girardot C, Gagneur J, Braun M, Furlong EE 2009 Combinatorial binding predicts spatio-temporal *cis*-regulatory activity. Nature 462: 65–70 19890324 10.1038/nature08531

